# Status of female sexual dysfunction among postmenopausal women in Bangladesh

**DOI:** 10.1186/s12905-022-01991-9

**Published:** 2022-10-04

**Authors:** Mohammad Ashraful Amin, Nusrat-E Mozid, Sanjana Binte Ahmed, Shakila Sharmin, Imran Hossain Monju, Shirin Shahadat Jhumur, Wharesha Sarker, Koustuv Dalal, Mohammad Delwer Hossain Hawlader

**Affiliations:** 1 Department of Public Health, North South University, Dhaka, 1229 Bangladesh; 2 Public Health Professional Developmental Society (PPDS), Dhaka, 1205 Bangladesh; 3 Division of Public Health Science, Institution for Health Sciences, Mid Sweden University, Sundsvall, Sweden

**Keywords:** Hormone therapy, Physical activity, Postmenopausal women, Female sexual dysfunction

## Abstract

**Background:**

Women's sexual health and physical desire for sex are most important for their emotional and physical well-being. This study aimed to examine the status of sexual dysfunction among postmenopausal women in Bangladesh and assess the significant risk factors behind this.

**Methods:**

A cross-sectional study was conducted among 45–55 years in four public and private hospitals in Bangladesh from April 2021 to June 2021 using a multi-stage sampling technique to enroll the study participants. The female sexual function index (FSFI) scale measured the prevalence of FSD, and the relationship of independent risk factors were assessed using a multivariate logistic regression model.

**Results:**

The total score of FSFI among postmenopausal Bangladeshi women was 18.07 ± 8.51. Among 260 participants, the prevalence of FSD was 56.9%. Out of all the significant risk factors, increasing age, urban population group, multiparous, homemakers, duration of menopause, and postmenopausal women with no hormone therapy were significantly associated with FSD. In contrast, those with regular physical activity were protective of FSD.

**Conclusion:**

In conclusion, a significant proportion of postmenopausal Bangladeshi women are enduring sexual dysfunction. Proper hormonal therapy and non-hormonal therapies such as physical activity and pelvic floor muscle (Kegel) exercise with adequate counseling are helpful to cope in this distressing situation.

## Introduction

Menopause is a transitory biological phenomenon, with significant meaning in women's lives [1]. It commonly occurs in midlife while most women are considerably fit and active [2]. The natural menopausal transition most often begins between ages 45 and 55, and the duration can depend on genetic, cultural, lifestyle, and dietary factors [3]. Women at their postmenopausal stage experience bio-psychosocial alterations, vaginal dryness and severe dyspareunia that prevent sexual engagement [4–6]. A significant numbers of women at their postmenopausal stage report having sexual difficulties, due to vaginal dryness and dyspareunia, lack of interest in sex or trouble in arousal [7, 8].

Female sexual dysfunction (FSD) is one of the most common health concerns; any sexual health problem causing distress to a woman is considered sexual dysfunction. It can be evaluated by knowing whether a woman is sexually active or has any problems with arousal, orgasm, and pain with sexual activity [9]. The incidence of these sexual issues rises as menopause draws near, peaking in the postmenopausal years [10]. Sexual dysfunction is related to low contentment, marital dissatisfaction, job loss, causing constant fatigue and stress [11]. These fundamental elements need consideration while providing specialized solutions during post-menopause [12]. As per community-based research, an estimated 68–86.5% of postmenopausal women face sexual dysfunction, harming their physical and psychological health [13]. This study employed FSFI-19, a gold standard and most frequent tool in assessing female sexual function as its domains or subscales allow it to examine the multidimensional character of sexual function [14].

The female life expectancy in Bangladesh is growing, reaching 73 years, and the mean menopausal age is 51.14 years [15]. Numerous postmenopausal women are expected to spend a significant portion of their existence. Female sexual dysfunction is still underexplored in Bangladesh. Women can experience sexual function problems such as reduced or absent interest in sexual activity, reduced or absent excitement or pleasure, reduced or absent arousal in the context of any sexual cues, delay or absence of orgasm at some point in their life [16]. Nevertheless, there is a substantial gap in understanding this particular community's sexual health issue. Hence, this study explored the status of sexual dysfunction among postmenopausal women in Bangladesh and assessed the significant risk factors behind this.

## Methods

**Fig. 1:**
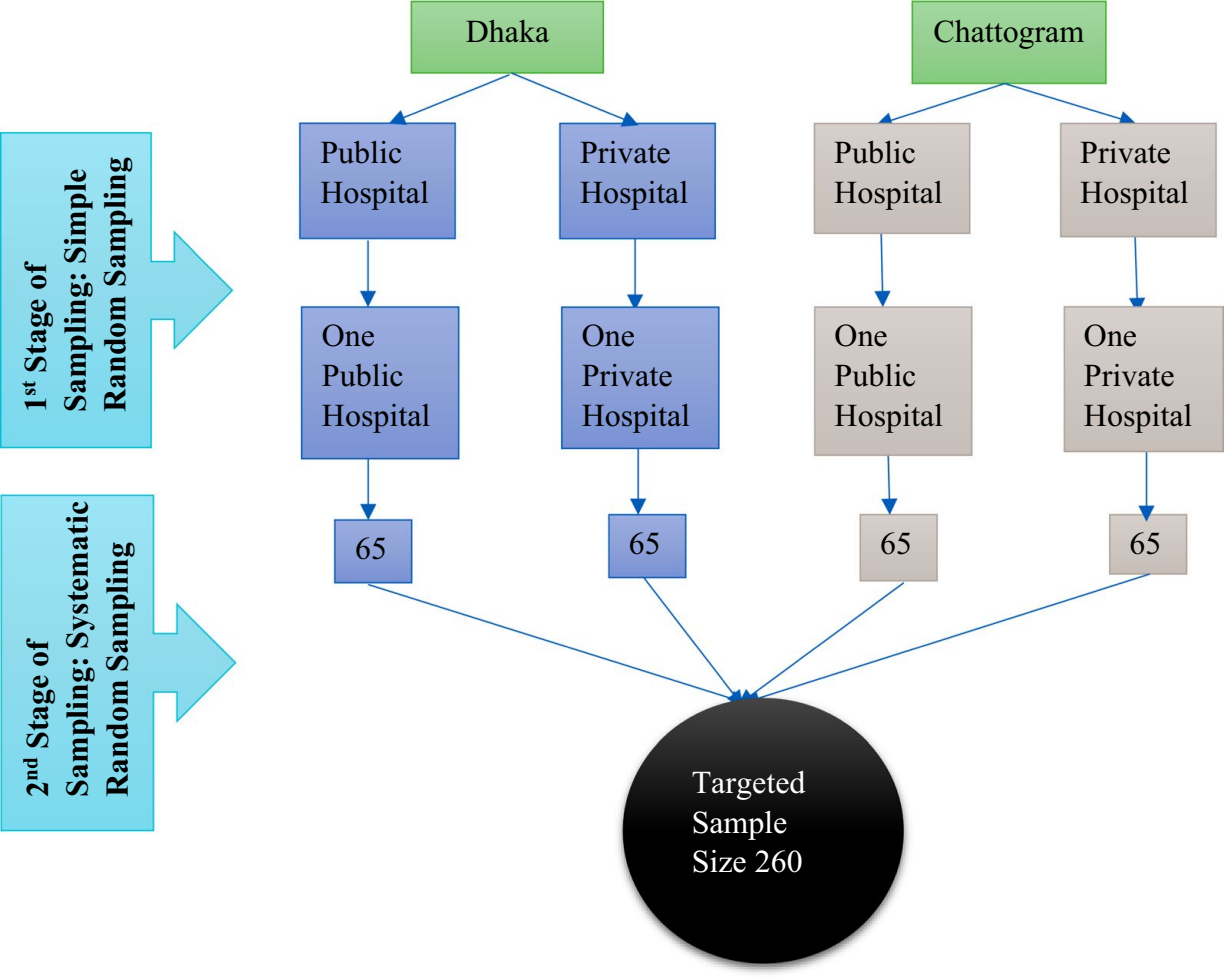
Multistage sampling technique

This cross-sectional study was conducted from April 2021 to June 2021 among postmenopausal women aged 45–55 years in Bangladesh. We excluded women who were divorced and widowed from this study. A multistage sampling technique was performed for collecting the data (Fig. 1). Dhaka and Chattogram districts were chosen as the study site. Four public and private medical hospitals were selected; two in the capital of Dhaka city (urban and rural area), two in Chattogram district (urban and rural area) by using a simple random sampling technique. The selection criterion was each hospital outpatient department size (n > 50) to ensure that sufficient data could be collected in a single visit, as the target sample size was drawn by systematic random sampling technique. Considering 80% power and 95% CI (0.05 to 1.96), the required sample size was 260.

Data was collected using a structured questionnaire by conducting a face-to-face interview. The questionnaire was first constructed in English, then translated into Bangla, and again back-translated to English by a third person to see the accuracy of the original Bangla translation. After each interview, data were checked for completeness and accuracy by a senior researcher. A team of well-trained physician-researchers with postgraduate education and public health training were involved in data collection. Regarding a sensitive issue amongst the Bangladeshi population, a practical training session was arranged for two days before starting data collection. We provided training about the questionnaire approach, maintaining ethical issues concerning social stigma. A pilot study was conducted among 20 participants from a public medical hospital in Dhaka city in a separate sample. The Cronbach's alpha coefficient was found to be 0.962, which showed excellent internal consistency of the questionnaire. The face validity had also done by consulting with experts in the fields. Participants were assured of keeping their information strictly confidential. Interviewers acquired written informed consent from the participants. For participants without formal education interviewers informed and explained them as per ethical guidelines and then they obtained informed consent signed by the participant's legally authorized representative. Participation in this study was entirely voluntary, no one was forced, and participants were allowed to leave any time if they felt uncomfortable answering any questions.

The questionnaires included participants' characteristics with personal details (age of menstrual cessation, duration of menopause, previous contraception uses for family planning, hormonal replacement therapy (HRT) intake, familiar with pelvic floor muscle exercise, physical activity). Based on the researchers' experience, the respondent's records accumulated seven significant diseases, hypertension, diabetes, chronic kidney disease (CKD), asthma, cancer, polycystic ovary syndrome (PCOS), and hyperprolactinemia. Trained female hospital staff conducted anthropometric measurements for estimating BMI. Women in their postmenopausal years were included wearing light indoor clothes, without shoes, and empty pockets whenever necessary. The individual's height was measured by gazing straight ahead and attaching their heels to a wall-mounted stadiometer precise to the closest 0.1 cm (Leicester Tanita HR 001). Tanita HA 503 weight scale, Tanita Corporation, Tokyo, Japan, was used to calculate the closest 0.1 kg weight. BMI was determined by multiplying weight in kilos divided by height squared in meters. In this formula (BMI = weight/height^2). BMI values were estimated as normal/healthy (between 18.5 and 24.9), overweight (between 25 and 29.9), and obese (30.0 or higher).

Postmenopausal women's last four weeks' sexual history was assessed to see the sexual function. The Female Sexual Function Index (FSFI)-19 item, a multidimensional self-reported questionnaire providing scores on six sexual function domains (desire, arousal, lubrication, orgasm, satisfaction, and pain), was used. Each of the domain's scores ranged from 0 (or 1) to 5. All six domains were multiplied by a homogenization factor and, the total FSFI-19 score was the sum of each domain. Validity test for the mean values of each domain and total FSFI-19 shows excellent internal consistency reliability co-efficient (0.949–0.965), and a high-test correlation was evaluated by applying intraclass correlation coefficient (ICC) to the full scale of FSFI-19 (0.809) (Table 2).

**Fig. 2:**
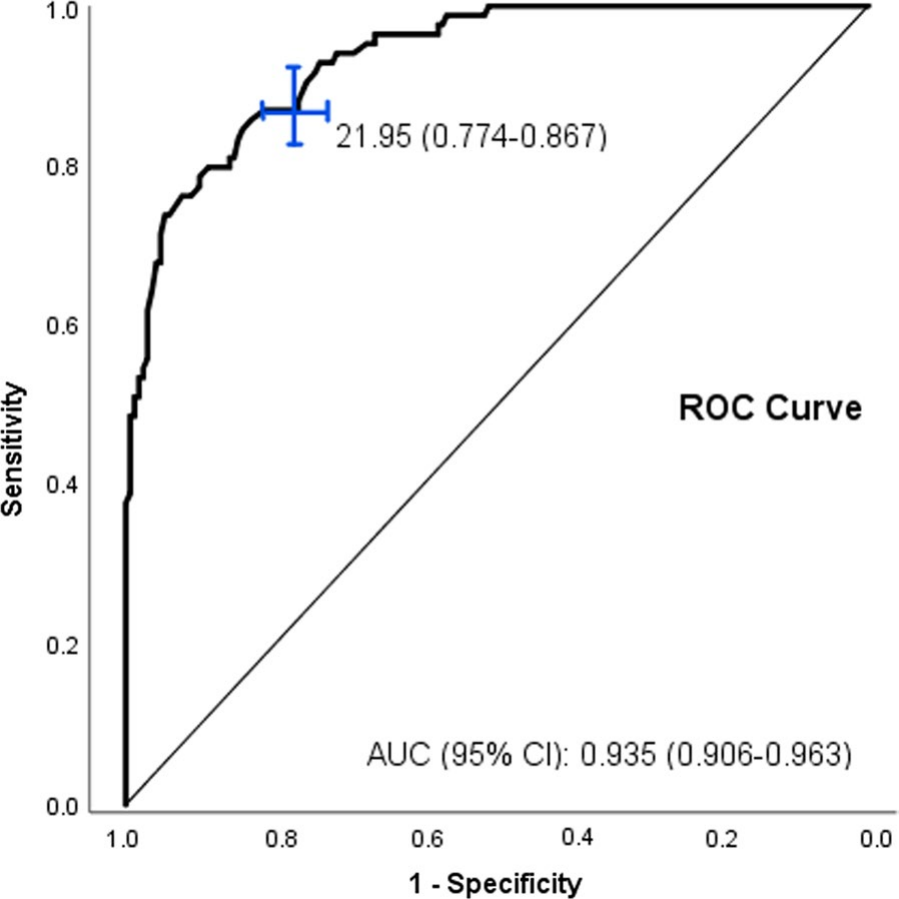
ROC curve; total FSFI scale

The concept of sexual dysfunction and sexual health-related personal distress is paramount to diagnosing all female sexual dysfunctions (FSD) [17]. Hence postmenopausal women's inventory of FSFI scale validation was a primary concern. In our study, the sexual health-related personal distress was not measured. However, the data on the sexual satisfaction domain of the FSFI was found lower than normal (54.9%), as evidence of how bothersome the dysfunction seems to have been among postmenopausal women. The FSFI scale was first developed by Rosen et al. in 2000 [18]. Since then, numerous studies have translated FSFI into many languages and used different cutoff scores to measure female sexual dysfunction. In Asian countries, the cutoff score ranged from 17.5 to 28.0 [19–22], whereas in North America or European countries, the score varied between 26.0 and 27.5 [23–26]. The current study determined the cutoff score of 21.95 following the FSFI score with the postmenopausal women in the diagnosis group from each domain. The inventory cutoff point was assessed using the receiver operating characteristics (ROC) curve and the area under the ROC curve (AUC). The cutoff point was 21.95 meaning any woman with a score lower than 21.95 was considered having female sexual dysfunction (FSD). This value represents a specificity of 77.4% and a sensitivity of 86.7%; the area under the curve (AUC) was found to be [0.935 (CI 0.906–0.963), *p* value < 0.01)] (Fig. 2).

Data were analyzed using Statistical Package for the Social Sciences (SPSS) software version 25.0. Descriptive statistics were used to calculate the frequencies, percentages, mean and standard deviation of all independent variables, FSFI domain score, and total score. The outcomes measured were reported as the prevalence of FSD. Additionally, Pearson (r) correlational coefficients were stated as evidence of concurrent validity for each domain and full FSFI scale. A multivariate logistic regression analysis was employed to adjust all significant independent variables as covariates in the fitted model. Hosmer–Lemeshow goodness of fit test showed model fitness. Logistic regression co-efficient from the model were exponentiated and presented as adjusted odds ratios (AOR) with a corresponding 95% confidence interval, with *p* value < 0.05 statistically significant. We also obtained logistic regression models' tolerance and variance inflation factors (VIF) to evaluate potential multicollinearity.

## Results

Three hundred and thirty-six participants expressed an interest in the study, and 269 gave consent to participate. After analyzing and checking for consistency verification and reduction, 260 data were included in the subsequent analysis with a response rate of 77.38%. The mean age of the postmenopausal women was 51.3 years, where 61.5% belong to the age group of 51–55 years. More than half of our participants were from urban areas (59.6%), and 62.7% of women had 2–4 children. 36.2% of women had a graduate degree, and the majority (46.5%) of our participants were homemakers. 47.7% of women were overweight. The mean age of menstrual cessation was 46.3 (in years). Amongst all our study participants, 53.5% were experiencing menopause for 2–5 years. Almost 91.5% of our participants had used contraceptives for family planning purposes. 37.3% of them had started or used HRT, which can be highly beneficial after menopause. We asked our participants whether they were familiar with the term pelvic floor muscle exercise or not, and only 19.2% responded positively. In terms of physical exercise, 44.2% of women had never had any physical activity in their lifetime. We recorded seven significant diseases from the investigation profile; where 20.4% were hypertensive, 29.6% were diabetic, 10.4% with bronchial asthma, 4.2% with chronic kidney disease, 1.5% with cancer, 3.5% with PCOS, and 1.5% with hyperprolactinemia (Table 1).

**Table 1:** Participant characteristics (n = 260)

Characteristics	Frequency (n)	Percentage (%)
*Age groups, years*		
45–50	100	38.5
51–55	160	61.5
Mean (SD)	51.3 (2.8)	
*Area of residence*		
Rural	105	40.4
Urban	155	59.6
*Number of children*		
One	63	24.2
Two–Four	163	62.7
> Four	26	10.0
None	8	3.1
*Education*		
No formal education	27	10.4
Up to class 8	29	11.2
Class 9–12	53	20.4
Graduation	94	36.2
Postgraduation	57	21.9
*Profession*		
Homemakers	121	46.5
Govt. Employee	62	23.8
Private Service	66	25.4
Business	11	4.2
*BMI (kg/m*^*2*^*)*		
Normal	119	45.8
Overweight	124	47.7
Obese	17	6.5
*Age of menstrual cessation, years*		
≤ 45	88	33.8
46–50	165	63.5
51+	7	2.7
Mean (SD)	46.3 (2.7)	
*Duration of menopause*		
1 year	26	10.0
2–5 years	139	53.5
6+ years	95	36.5
*Previous contraception uses for family planning*		
No	22	8.5
Yes	238	91.5
*Hormonal replacement therapy intake*		
No	163	62.7
Yes	97	37.3
*Familiar with pelvic floor muscle exercise*		
No	210	80.8
Yes	50	19.2
*Physical activity*		
Daily	34	13.1
2–3 times per week	75	28.8
Once a week	36	13.8
Rarely/never	115	44.2
*Any existing disease*		
HTN (+)	53	20.4
Diabetes (+)	77	29.6
Cancer (+)	4	1.5
Asthma (+)	27	10.4
CKD (+)	11	4.2
PCOS (+)	9	3.5
Hyperprolactinemia (+)	4	1.5

SD = standard deviation; HTN = hypertension; CKD = chronic kidney disease; PCOS = polycystic ovarian syndrome

The mean and standard deviation of all six domains (Desire, Arousal, Lubrication, Orgasm, Satisfaction, Pain) and the total score of the FSFI had presented in (Table 2). The mean score of FSFI was 18.07 ± 8.51 (mean ± SD). Among six domains, the lowest score was arousal (2.65 ± 1.46), and the highest score was satisfaction (3.42 ± 1.52). The prevalence of FSD was 56.9%. This high prevalence implies the deplorable status of postmenopausal women in Bangladesh.

**Table 2:** The mean, SD and reliability of the FSFI domains and total Scores

FSFI domains	Mean ± SD	Score range	α	ICC
Desire	3.17 ± 1.32	1.2–6.0	0.965	
Arousal	2.65 ± 1.46	0.0–6.0	0.949	
Lubrication	2.96 ± 1.63	0.0–6.0	0.950	
Orgasm	2.79 ± 1.59	0.0–6.0	0.948	0.809
Satisfaction	3.42 ± 1.52	0.8–6.0	0.958	
Pain	3.10 ± 1.74	0.0–6.0	0.959	
FSFI total score	18.07 ± 8.51	2.0–36.0	0.962	

Cronbach’s α coefficients showed excellent internal consistency reliability of the total and domain scores of the FSFI. ICC showed good reliability for the individual domains as well as to the full scale of the FSFI

SD: standard-deviation; α: Cronbach’s alpha; ICC: intra-class correlations coefficient

In Table 3, the concurrent validity for all the domains and full FSFI-19 inter-correlations had shown significant correlation ranged from r = 0.628 to r = 0.921 (*p* value < 0.001).

**Table 3:** Domain inter-correlations of the FSFI questionnaire

	Desire	Arousal	Lubrication	Orgasm	Satisfaction	Pain
Desire	–					
Arousal	0.845**	–				
Lubrication	0.773**	0.921**	–			
Orgasm	0.768**	0.913**	0.900**	–		
Satisfaction	0.674**	0.795**	0.794**	0.865**	–	
Pain	0.628**	0.822**	0.845**	0.843**	0.800**	–

**Correlation is significant at the 0.01 level (2-tailed)

The relationship between independent risk factors for FSD among postmenopausal women was performed by multivariable logistic regression analysis as presented in Table 4. Among all the factors, the age group 45–50 years were 32% less likely towards FSD than the 51–55 years (OR = 0.68, CI 0.41–1.12). The rural population group was 85% less likely towards FSD than the urban population group (OR = 0.15, CI 0.08–0.27). Women with more than four children showed 1.83 times higher risk towards FSD comparing others (OR = 1.83, CI 0.27–12.54). Homemakers were 4.61 times higher odds towards FSD (OR = 4.61, CI 1.30–16.34). Women with a duration of menopause of one year were 73% less likely towards FSD than those with two-five years and above (OR = 0.27, CI 0.11–0.67). Hormonal replacement therapy (HRT) also showed a significant association with female sexual function. Postmenopausal women with no HRT use were 5.42 times more likely to have FSD (OR = 5.42, CI 3.14–9.35). Low physical activity is related to female sexual dysfunction. In our study, women who were physically active daily in their life showed 89% less likely risk towards FSD in comparison to others (OR = 0.11, CI 0.05–0.25).

**Table 4:** Relationship of independent risk factors for FSD using multivariate logistic regression

Risk factors	Adjusted OR	95% CI	
*Age groups, years*			
45–50	0.68	0.41–1.12**	
51–55	Reference		
*Area of residence*			
Rural	0.15	0.08–0.27**	
Urban	Reference		
*Number of children*			
One	0.23	0.04–1.25	
Two-Four	0.45	0.09–2.32	
> Four	1.83	0.27–12.54**	
None	Reference		
*Education*			
No formal education	5.32	5.35–16.84	
Up to class 8	7.00	5.79–25.73	
Class 9–12	4.24	1.91–9.40	
Graduation	1.55	0.78–3.07	
Postgraduation	Reference		
*Profession*			
Homemakers	4.61	1.30–16.34**	
Govt. Employee	0.87	0.24–3.15	
Private Service	0.56	0.15–2.04	
Business	Reference		
*BMI (kg/m*^*2*^*)*			
Normal	1.51	0.50–4.57	
Overweight	2.32	0.77–6.98	
Obese	Reference		
*Duration of menopause*			
1 year	0.27	0.11–0.67**	
2–5 years	0.61	0.36–1.05	
6 + years	Reference		
*Previous contraception uses for family planning*			
No	8.59	1.97–37.59	
Yes	Reference		
*Hormonal replacement therapy intake*			
No	5.42	3.14–9.35**	
Yes	Reference		
*Physical activity*			
Daily	0.11	0.05–0.25**	
2–3 times per week	0.12	0.06–0.23**	
Once a week	0.20	0.09–0.45**	
Rarely/never	Reference		
*Any existing disease*			
HTN	No	0.68	0.32–1.44
	Yes	Reference	
Diabetes	No	0.28	0.15–0.55
	Yes	Reference	
Cancer	No	-	-
	Yes		
Asthma	No	1.87	0.74–4.71
	Yes	Reference	
CKD	No	-	-
	Yes		
PCOS	No	3.13	0.52–11.45
	Yes	Reference	
Hyperprolactinemia	No	3.54	0.27–31.65
	Yes	Reference	

AOR = adjusted odds ratio; CI = confidence interval; HTN = hypertension; CKD = chronic kidney disease; PCOS = polycystic ovarian syndrome

**p < 0.01

## Discussion

Sexual dysfunction is a prevalent problem with the sexual response cycle that interferes with regular, satisfying sexual activity. There is a dearth of literature querying FSD in low- and middle-income countries. Considering the taboo and sensitive cultural issues of FSD, our study from Bangladesh could be an essential contribution to the field. Our study has some noteworthy findings from Bangladesh context. Several risk factors were strongly associated with sexual dysfunction of the postmenopausal women in Bangladesh: advancing age (51–55 vs 45–50), urban vs rural, more than 4 children, homemaker vs employed; longer duration since menopause (2–5 years vs 1 year), no hormonal replacement therapy vs hormonal replacement therapy, and low physical activity.

Socio-demographic characteristics, cultural beliefs, happy marriages, or relationships can alter the prevalence rate of FSD. Epidemiologic research in FSD, particularly in clinical populations, found that the estimated prevalence rate was 43% in a US population aged 18–59 [27]. According to a study performed in Italy, 40% of women presented with more than one sexual function problem [28]. By contrast, Asian countries investigating FSD demonstrated a wide range of prevalence rates. In Malaysia, it was 5.5% [29], whereas the value was 46.1% in Ansan, Korea [30]. FSD was significantly higher in the middle eastern region. A study from Jordan and Iran found 64.7% and 84.33% of their study population with FSD, respectively [31, 32]. Different results demonstrate that women's sexual health and sexual function require attention.

Increasing age had been a concern among Turkish women where 46–55 years age group presented the highest prevalence rate of FSD, 67.9% [33]. Postmenopausal women residing in a rural area were linked to having better sexual function from a study in Granada, Spain [34]. Apart from that, multiparity is a significant risk factor for FSD. According to a population-based study performed in Finland, nulliparous women had significant sexual pain difficulties and were sexually less pleased despite the number of children [35]. While in Iran and Palestine, multiparity was significantly associated with FSD [36, 37]. It is evident from many studies that some factors had unclear effects on FSD, such as race, education, employment, parity, being in a relationship, frequency of sexual intercourse, smoking, and alcohol consumption [38]. Thereby a study from Singapore, hospital nurses were less likely to develop FSD [39], and employed women were considerably more likely to present with vaginismus than unemployed women [40]. In Malaysia and Iran, women who received education up to secondary school and homemakers were more at risk of FSD [41, 42].

How long menopausal symptoms last is individual to each woman, but research has shown that menopausal symptoms last an average of 4.5 years following a woman’s last period. Healthcare professionals advise people to expect a 7-year duration for their symptoms [43]. During this stage, menopausal symptoms tend to subside but may continue for an average of four to five years. Hormone levels subside, which leads to FSD impacting quality of life [44]. In Malaysia, menopausal status was related to FSD 6.6 times [45], which also was found in midlife Singaporean women [46]. When a woman starts experiencing menopausal symptoms, the general practitioner usually prescribes HRT with a low dose. Menopausal hormone therapy (MHT) improves vaginal dryness, hot flushes, and night sweats, along with many other menopausal symptoms [47]. It can be advantageous up to 60 years, and based on their physical wellbeing; women can continue it after that age [48, 49]. Estrogen is known as the "female" hormone, which promotes the growth and health of the female reproductive organs and keeps the vagina moisturized. Reduced estrogen production affects sexual function directly, and regular intake of HRT can improve that. Different studies have focused on its role, which showed its effect on fasting plasma glucose levels, low-density lipoprotein, and total cholesterol in peri- and postmenopausal women [50]. HRT enhances sexual function in a statistically significant way; hence the level of sexual satisfaction is better in postmenopausal women with HRT than those without HRT [51]. Our study has supported the existing knowledge of effectivity of HRT for FSD among women in Bangladesh, which has immense significance for literature using a context of low-income country.

Physical activity was a protective risk factor in our study. Similarly, in Iran and Brazil, sedentary women had a greater frequency of FSD compared to active and moderately active women [36, 52]. Women with regular exercise scored higher in FSFI domains with low sexual distress in Italy [53]. Physical activity stimulates the autonomic nervous system, increasing genital blood flow and arousal, daily at least 30 min of exercise could boost sexual arousal [54]. Pelvic floor muscle (Kegel) exercise plays an essential role in improving sexual dysfunction [55]. Strengthening pelvic muscles reduce sexual pain and increase the ability to pleasure [56]. During pregnancy, many physicians recommend it, as pelvic floor muscles support the baby and assist in the painless delivery [57]. Nonfunctional pelvic floor muscle had more impaired sexual function in Brazilian menopausal women [58]. Maintaining a healthy pelvic floor increase sexual interest and help with arousal problems [59]; its beneficial effects have also been observed in postmenopausal women in China [60]. Among post-partum women with dyspareunia after delivery showed pelvic floor myofascial therapy improves pelvic floor muscle function, and exercise had positive effects on better sexual function [61]. Like other Asian countries, this term is unfamiliar to us; physicians could introduce pelvic floor muscle exercise and its benefit among the Bangladeshi population.

The selection criteria of our participants within a hospital setting were random; therefore, it does not show any selection bias. Another strength was using the FSFI-19 specific items and validated scales of each domain to assess the prevalence. However, a larger sample size covering every district could reflect the study result. Some potential confounders were not addressed in our studies, such as stability of the relationship, happy marriage or relationships, smoking and alcohol consumption history, sexually Transmitted Diseases, and psychiatric illness. We tried to cover several significant non-communicable diseases, including common reproductive health concerns for women, for example, PCOS and endocrine disorder hyperprolactinemia. Although we did not find any relationship with FSD, we recommend other studies to cover some clinical health issues such as prior pelvic surgery, endometriosis, uterine fibroids, and the information on whether the presence of erectile dysfunction in a male partner could be a vital factor for female sexual dysfunction. We have considered only seven diseases in the risk factor. However, in a larger study we recommend to including more communicable and non-communicable diseases including family violence and injuries.

## Conclusion

Compared with many other Asian countries, we found that the rate of FSD among postmenopausal Bangladeshi women was substantially higher. It offers a broad picture and serves the evidence of the sexual health needs in Bangladesh. Among several potential risk factors increasing age, the number of children, profession, and duration of menopause was significant. On the contrary, hormone replacement therapy, physical exercise, and pelvic floor muscle exercise were found to have a protective effect on FSD. Satisfying sex life is vital to a woman's wellbeing at every age. FSD is treatable; identifying the underlying condition, lifestyle changes, hormone therapy, being physically active has profound benefits. It is crucial to consult physicians and health care providers on this occasion without shame, guilt, or fear. Rather than considering sexual issues taboo, it needs to be openly discussed and portrayed on television, magazines, and the internet to grow awareness and understanding of sexual health problems. The outlook for sexual problems can improve with counseling, education, and communication.

## Data Availability

The data underlying the results presented in this study will be provided on reasonable request to Dr. Mohammad Delwer Hossain Hawlader. Email: mohammad.hawlader@northsouth.edu.

## Abbreviations

CKD: Chronic kidney disease
FSD: Female sexual dysfunction
FSFI: Female sexual function index
HRT: Hormonal replacement therapy
HTN: Hypertension
ICC: Intraclass correlation coefficient
MHT: Menopausal hormone therapy
PCOS: Polycystic ovarian syndrome
ROC: Receiver operating characteristics
